# One new genus and two new species of oonopid spiders from Xishuangbanna Rainforest, southwestern China (Araneae, Oonopidae)

**DOI:** 10.3897/zookeys.494.9183

**Published:** 2015-04-06

**Authors:** Yanfeng Tong, Shuqiang Li

**Affiliations:** 1 College of Chemistry and Life Science, Shenyang Normal University, Shenyang 110034, China; 2 Institute of Zoology, Chinese Academy of Sciences, Beijing 100101, China

**Keywords:** Taxonomy, goblin spider, diagnosis, morphology, tropical forest

## Abstract

A new genus, *Bannana*, is established for two new species that resemble those of the *Dysderoides* complex. Two new species are described, *Bannana crassispina* **sp. n.** and *Bannana parvula* **sp. n.** Morphological descriptions and illustrations of both new species are given.

## Introduction

The “*Dysderoides* complex”, including the genera *Dysderoides* Fage, 1946, *Himalayana* Grismado, 2014 and *Trilacuna* Tong & Li, 2007, was firstly proposed by Grismado et al. (2014). This Asian genera complex has a wide distribution, from Pakistan to Sumatra, and sharing the general morphology of the genitalia, chelicerae and labium.

When examining specimens collected from leaf litter in Xishuangbanna, Yunnan Province of China, two new species were recognized. They are very similar to those species of *Dysderoides*, having reduced eyes, deeply incised labium and complicated male palpal bulb, but without macrosetae on legs III and IV. Here a new genus belonging to the *Dysderoides* complex is established to accommodate these two new species.

## Material and methods

Specimens in this study were mainly collected by pitfall-trapping and hand-collecting from leaf-litter in tropical rainforest in Xishuangbanna, Yunnan in 2006 and 2007. All specimens are deposited in the Institute of Zoology, Chinese Academy of Sciences in Beijing (IZCAS).

The specimens were examined using a Leica M205C stereomicroscope. Details were studied with the use of an Olympus BX51 compound microscope. All illustrations were made using a drawing tube and inked on ink jet plotter paper. Vulvae were cleared in lactic acid. Photos were made with a Canon EOS 550D zoom digital camera (18 megapixels). Images from multiple focal planes were combined using Helicon Focus (version 3.10) image stacking software. Descriptions were generated with the aid of the Planetary Biodiversity Inventory descriptive goblin spider database and shortened where possible. Measurements were taken using an Olympus BX51 compound microscope and are in millimeters.

## Taxonomy

### Family Oonopidae Simon, 1890

#### Bannana gen. n.

Taxon classificationAnimaliaAraneaeOonopidae

http://zoobank.org/108014DB-D372-49AF-974D-D412DD02E18E

##### Type species.

*Bannana crassispina* sp. n.

##### Etymology.

The generic name is derived from the last a few letters of the type locality, ‘Xishuangbanna’, and is feminine in gender.

##### Diagnosis.

**Figure 1.**
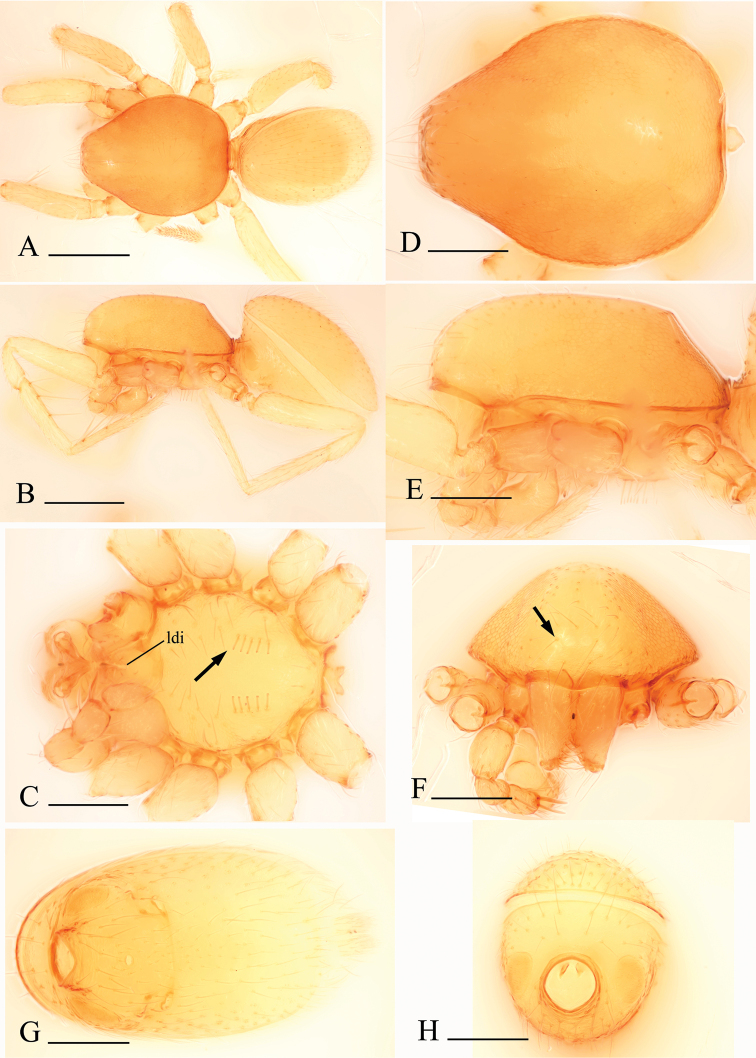
*Bannana crassispina* sp. n., male. **A, B** Habitus, dorsal and lateral views **C, D, E, F** Prosoma, ventral, dorsal, lateral and anterior views (arrows show the regular setae in Fig. C and the reduced eyes in Fig. F) **G, H** Abdomen, ventral and anterior views. Abbreviation: ldi = labium deep incision. Scales bar: **A, B** = 0.4 mm; **C–H** = 0.2 mm.

**Figure 2.**
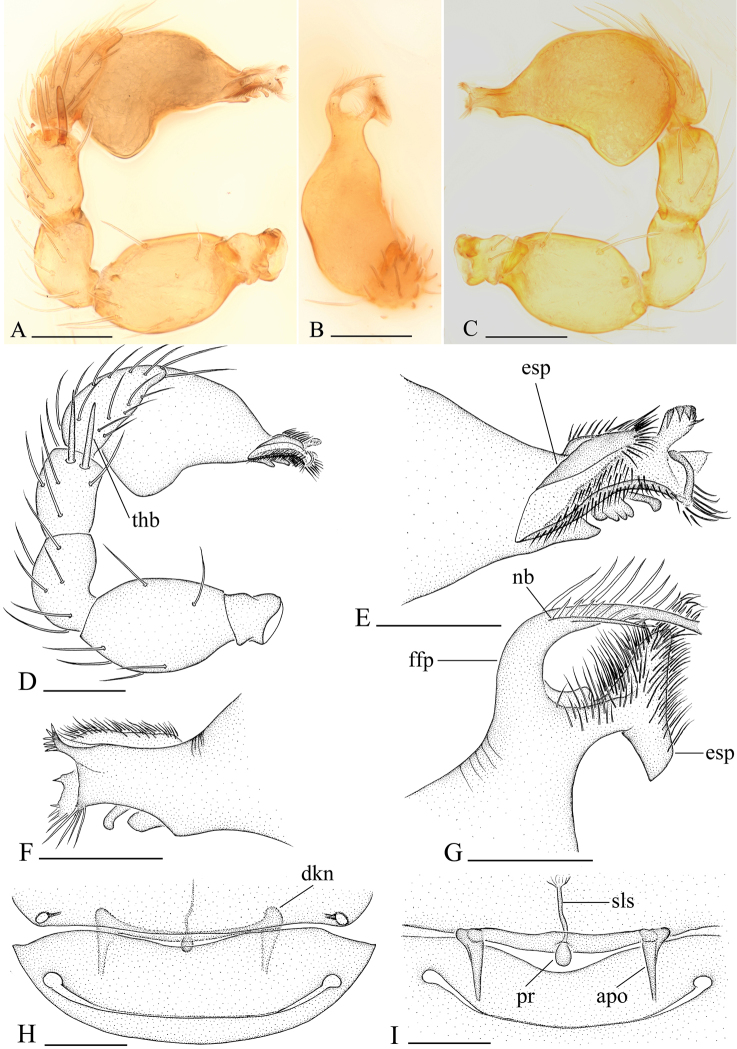
*Bannana crassispina* sp. n., **A–G** male **H, I** female **A, C, D** Left palp, prolateral (**A, D**) and retrolateral (**C**) views **B, E, F, G** Distal part of bulb, dorsal (**B, G**), prolateral (**E**) and retrolateral (**F**) views **H, I** Genital area, ventral and dorsal views. Abbreviations: apo = apodeme; dkn = dark brown knobs; esp = ear-shaped protrusion; ffp = filiform, curved projection; nb = narrow branch; pr = posterior receptacle; sls = stick-like sclerite; thb = thick bristles. Scales bar: **A–D, H, I** = 0.1 mm; **E–G** = 0.05 mm.

**Figure 3.**
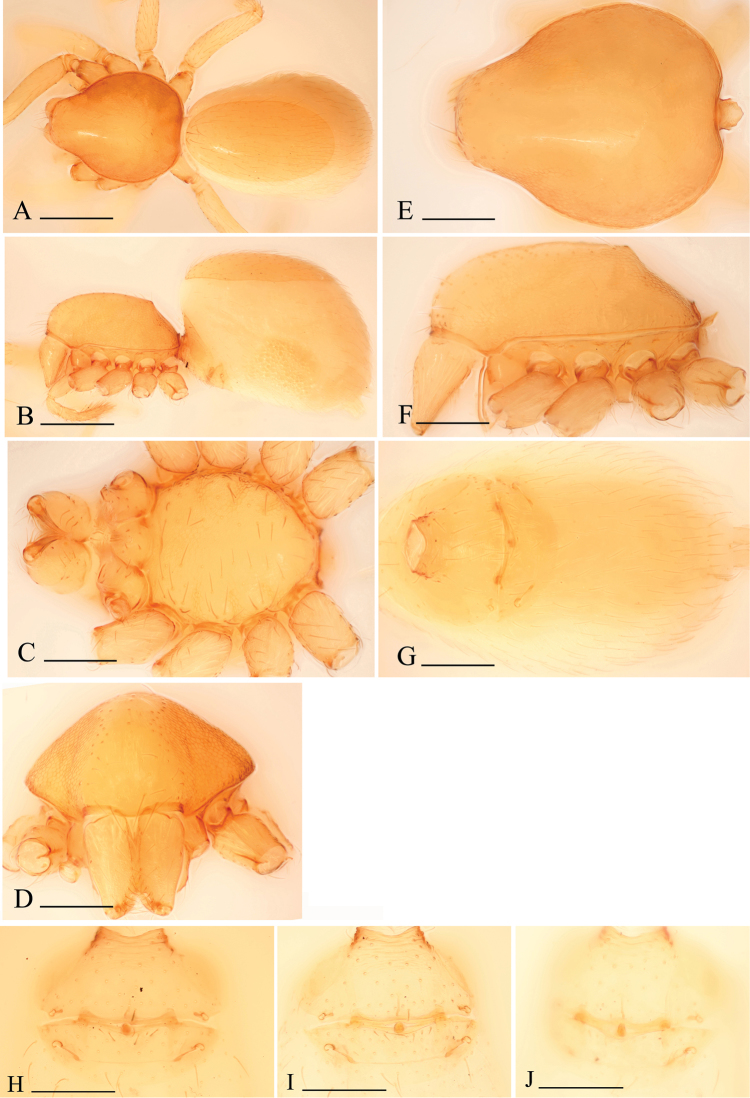
*Bannana crassispina* sp. n., female. **A, B** Habitus, dorsal and lateral views **C–F** Prosoma, ventral, anterior, dorsal and lateral views **G** Abdomen, ventral view **H–J** Genital area, ventral (**H, I**) and dorsal (**J**) views, **I, J** cleared in lactic acid. Scales bar: **A, B** = 0.4 mm; **C–J** = 0.2 mm.

**Figure 4.**
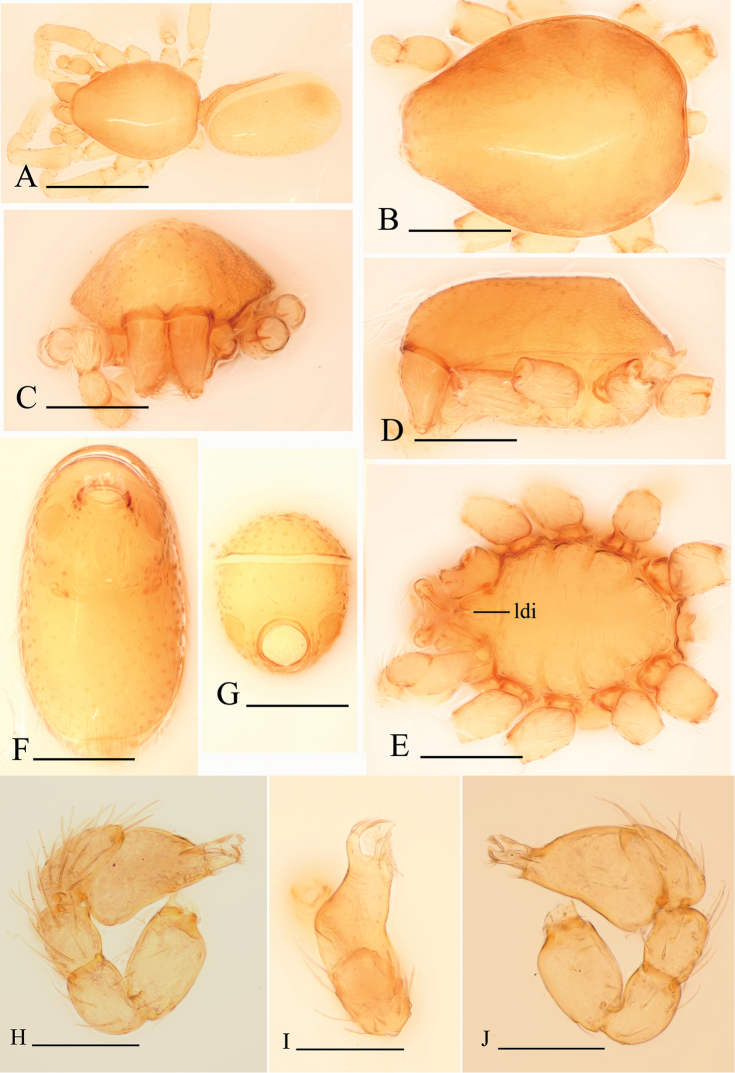
*Bannana parvula* sp. n., male. **A** Habitus, dorsal view **B, C, D, E** Prosoma, dorsal, anterior, lateral and ventral views **F, G** Abdomen, ventral and anterior views **H–J** Left palp, prolateral, dorsal and retrolateral views. Abbreviation: ldi = labium deep incision. Scales bar: **A** = 0.4 mm; **B–G** = 0.2 mm; **H–J** = 0.1 mm.

**Figure 5.**
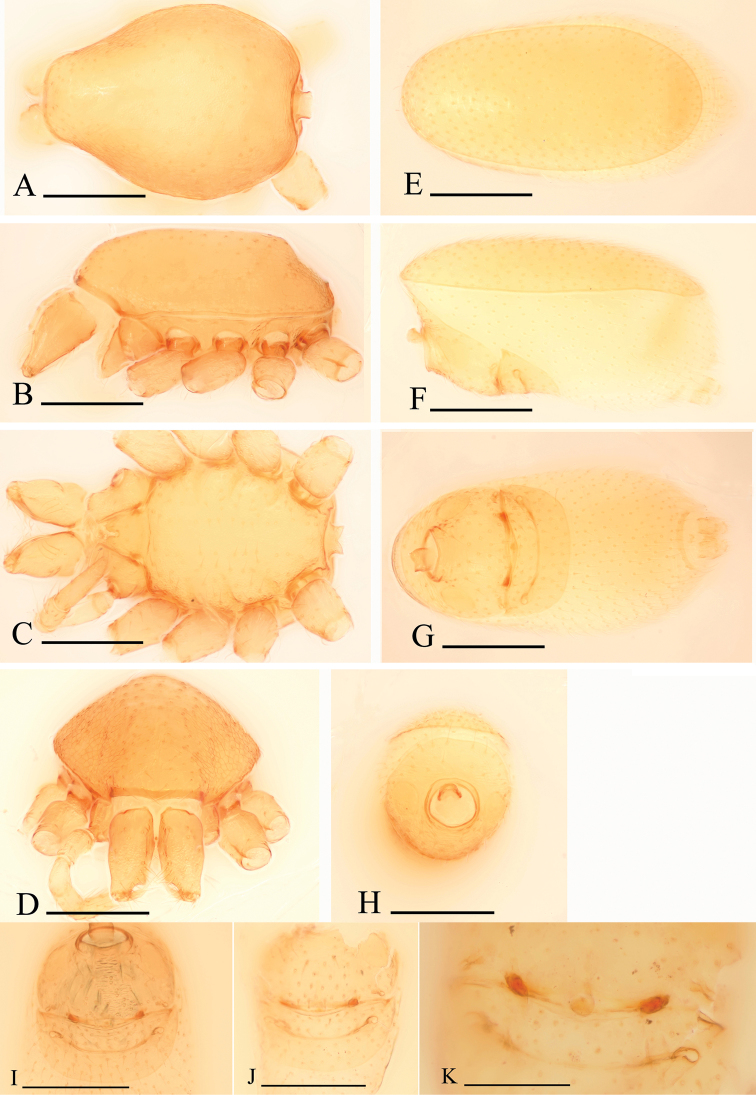
*Bannana parvula* sp. n., female. **A–D** Prosoma, dorsal, lateral, ventral and anterior views **E–H** Abdomen, dorsal, lateral, ventral and anterior views **I–K** Genital area, ventral (**I, J**) and dorsal (**K**) views **J, K** cleared in lactic acid. Scales bar: **A–J** = 0.2 mm; **K** = 0.1 mm.

**Figure 6.**
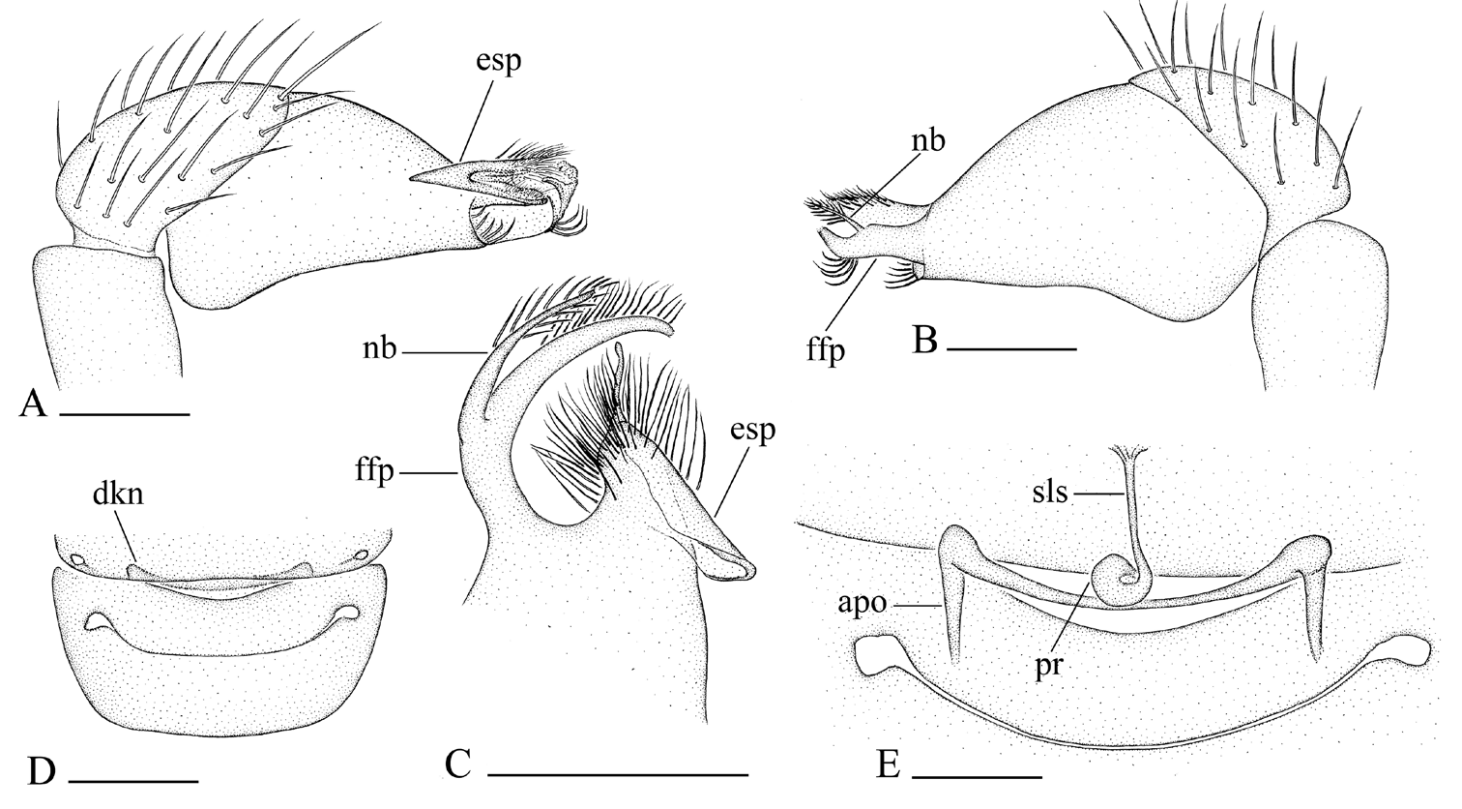
*Bannana parvula* sp. n. **A, B** Male bulb, prolateral and retrolateral views **C** Distal part of male bulb, dorsal view **D, E** Female genital area, ventral and dorsal views. Abbreviations: apo = apodeme; dkn = dark brown knobs; esp = ear-shaped protrusion; ffp = filiform, curved projection; nb = narrow branch; pr = posterior receptacle; sls = stick-like sclerite. Scales bar: **A, B, C, E** = 0.05 mm; **D** = 0.1 mm.

The new genus is similar to *Dysderoides* but can be distinguished from the latter by the following combination of characters: 1) lacking macrosetae on legs III and IV; 2) having reticulate cuticle on the sternum and the sides of the carapace (Figs 1F, 3D, 4C, 5D), which is smooth in *Dysderoides*; 3) with radial furrows between coxae I–II, II–III, III–IV on the sternum, which is absent in *Dysderoides*; 4) females have large dorsal scutum (Figs 3A, B, 5E, F), which is absent or less than half of dorsum in *Dysderoides*. The new genus can be easily distinguished from *Trilacuna* and *Himalayana* by the reduced eyes (Figs 1F, 3D, 4C, 5D) and the reticulate cuticle on the sides of the carapace. Both *Trilacuna* and *Himalayana* have normal eyes and usually granulated or sometimes smooth on the sides of the carapace (Eichenberger 2011; Grismado et al. 2014; Tong and Li 2007, 2013). The new genus also can be distinguished from *Trilacuna* by the short postepigastric scutum in females (Figs 3G, 5G) and by having a furrow connecting the posterior tracheal spiracles in males (Figs 1G, 4F); can be distinguished from *Himalayana* by the absence of the acute projection in the prolateral dorsal part of the male bulb (see Grismado et al. 2014: fig. 62D–H) and the straight, stick-like sclerite in female genital area (Figs 2I, 6E).

##### Description.

Male: cephalothorax: *carapace* yellow, without any pattern, broadly oval in dorsal view, pars cephalica slightly elevated in lateral view, anteriorly narrowed to 0.49 times its maximum width or less, with rounded posterolateral corners, posterolateral edge without pits, posterior margin not bulging below posterior rim, anterolateral corners without extension or projections, posterolateral surface without spikes, surface of elevated portion of pars cephalica smooth, sides reticulated, thorax without depressions, fovea absent, without radiating rows of pits; lateral margin straight, smooth, rebordered, without denticles; marginal setae present. Clypeus margin unmodified, sinuous in front view, vertical in lateral view, median projection absent; setae light, needlelike. Chilum absent. Eyes absent (remnants still visible in *Bannana crassispina* sp. n.). *Sternum*: longer than wide, with radial furrows between coxae I–II, II–III, III–IV, uniform, not fused to carapace, median concavity absent, surface reticulated, microsculpture covering entire surface, anterior margin unmodified, posterior margin not extending posteriorly of coxae IV, anterior corner unmodified, distance between coxae approximately equal, lateral margins unmodified, without posterior hump; setae sparse, dark, needlelike, evenly scattered, without hair tufts (*Bannana crassispina* sp. n. has pairs of short setae in central part, as in Fig. 1C). *Mouthparts*: chelicerae straight, anterior face unmodified; without teeth on both promargin and retromargin; fangs without toothlike projections, directed medially, shape normal, without prominent basal process, tip unmodified; setae light, needlelike, evenly scattered; paturon inner margin with pairs of enlarged setae, distal region abruptly narrowed, posterior surface unmodified, promargin unmodified. Labium triangular, anterior margin deeply incised (as in Fig. 1C), same as sternum in sclerotization, not fused to sternum. Endites distally not excavated, anteromedian tip unmodified, posteromedian part unmodified, same as sternum in sclerotization. Abdomen: ovoid, rounded posteriorly. Dorsal scutum covering whole dorsum, strongly sclerotized, without color pattern. Epigastric scutum strongly sclerotized, surrounding pedicel. Postepigastric scutum strongly sclerotized, long, almost rectangular, covering nearly full length of abdomen length, anterior margin unmodified, without posteriorly directed lateral apodemes. Book lung covers large, smooth, anterolateral edge unmodified. Scutopedicel region unmodified, scutum not extending far dorsal of pedicel, plumose hairs absent. Posterior spiracles connected by groove. Spinneret scutum present, incomplete ring. Spinneret scutum without fringe of setae. Legs: pale, without color pattern; femur IV not thickened, same size as femora I–III, patella plus tibia I longer than carapace. Leg spines: tibiae I, II with 3 or 4 pairs of ventral spines each; metatarsi I, II with 2 pairs of ventral spines each, legs III and IV without spines. Genitalia: epigastric region with sperm pore large, oval, rebordered, situated in front of or at level of anterior spiracles. Palp normal size, not strongly sclerotized, right and left palps symmetrical, proximal segments yellow-brown; embolus light; trochanter normal size, unmodified; femur enlarged, attaching to patella basally; patella shorter than femur, not enlarged, setae unmodified; tibia not enlarged, distal part with modified setae in *Bannana crassispina* (Fig. 2A, D); cymbium yellow-brown, narrow in dorsal view, not fused with bulb, not extending beyond distal tip of bulb; bulb 1.5 to 2 times as long as cymbium, tapering apically; distal part with several laminae that bear filiform projections surrounding the embolus.

Female: as in male except as noted. Palp without claw; spines absent. Abdomen: dorsal scutum large, covering more than 3/4 of dorsum (Figs 3A, 5E). Postepigastric scutum short, only around epigastric furrow, not fused to epigastric scutum (Figs 3G, 5G). Supraanal scutum absent. Postepigastric area setae needlelike. Genitalia: ventral view: without special external features; dorsal view: there are one transverse ventral plates, adjacent to a pair of short apodemes; posterior receptacle rounded to ovoid, extending anterior by a narrow, stick-like sclerite (Figs 2H, I, 6D, E).

##### Composition.

*Bannana crassispina* sp. n. and *Bannana parvula* sp. n.

##### Distribution.

China (Yunnan).

#### Bannana crassispina sp. n.

Taxon classificationAnimaliaAraneaeOonopidae

http://zoobank.org/052CF748-1DF3-4D4F-BE90-B50BAA36F86B

##### Type material.

**Holotype:** male (IZCAS Ar-25082), China: Yunnan Province, Mengla County, Menglun Nature Reserve, Secondary tropical seasonal moist forest (21°54.718'N, 101°16.940'E, Alt: 645 m), pitfall traps, 16–31 April 2007, G. Zheng and Z. Chen leg. **Paratypes:** 1 male (IZCAS Ar-25085), same data as holotype; 1 female (IZCAS Ar-25080), same data as holotype; 1 female (IZCAS Ar-25078), same data as holotype; 1 female (IZCAS Ar-25084), same locality as holotype, 16–31 March 2007; 1 female (IZCAS Ar-25083), same locality as holotype, 1–15 May 2007; 1 female (IZCAS Ar-25087), same locality as holotype, 1–15 May 2007; 1 female (IZCAS Ar-25077), same locality as holotype, 16–31 May 2007; 1 male (IZCAS Ar-25074), 21°54.607'N, 101°17.005'E, Alt: 633 m, pitfall traps, 16–31 May 2007; 2 males (IZCAS Ar-25073), same locality as above, searching, 4–11 May 2007; 1 female (IZCAS Ar-25075), 21°54.984'N, 101°16.982'E, Alt: 656 m, pitfall traps, 16–31 April 2007; 1 male (IZCAS Ar-25072), same locality as above, 16–24 November 2006; 1 male (IZCAS Ar-25076), same locality as above, 16–28 February 2007; 1 female (IZCAS Ar-25081), 16–31 May 2007; 1 female (IZCAS Ar-25086), 16–31 June 2007; 1 female (IZCAS Ar-25079), Secondary tropical seasonal rainforest (21°55.428'N, 101°16.441'E, Alt: 598 m), pitfall traps, 16–31 June 2007.

##### Etymology.

The specific name is Latin, “crass-” = thick, and “spin-” = seta, referring to the thick bristles on male palpal tibiae.

##### Diagnosis.

The males of the new species can be distinguished from *Bannana parvula* sp. n. by the thick bristles on palpal tibiae (thb in Fig. 2A, D) and rows of setae on the central part of sternum (Fig. 1C); females of the new species are similar to those of *Dysderoides synrang* Grismado & Deeleman, 2014, but can be distinguished by the absence of macrosetae on legs III and IV, and by the large dorsal abdominal scutum.

##### Description.

Male. Body yellow, legs lighter. Habitus as in Fig. 1A, B. Body length 1.47; carapace 0.75 long, 0.49 wide; abdomen 0.85 long, 0.48 wide. Carapace broadly oval, *pars cephalica* slightly elevated in lateral view, dorsal surface smooth; sides reticulated; lateral margin rebordered; eyes reduced, only four eyes visible in frontal view (Fig. 1F). Mouthparts: chelicerae straight, paturon inner margin unmodified; labium anterior margin deeply incised (ldi) (Fig. 1C); endites slender, distally only slightly branched. Sternum: setae sparse, light, needle-like, evenly scattered; on the middle part of sternum with five pairs of short setae arranged in two rows (Fig. 1C). Abdomen: dorsal scutum covering full length of abdomen, no soft tissue visible from above, not fused to epigastric scutum. Pedicel tube short, unmodified. Book lung covers elliptical, surface smooth. Postepigastric and epigastric scutum fused, apodemes absent, posterior spiracles connected by groove (Fig. 1G). Leg spines: tibiae I, II with 4 pairs of ventral spines each; metatarsi I, II with 2 pairs of ventral spines each, legs III and IV without spines.

Male genitalia: epigastric region (Fig. 1G) with sperm pore small, oval, rebordered, situated in front of anterior spiracles. Palp (Fig. 2A–G): pale-orange; femur enlarged, attached to patella basally; tibia with two very strong, thick bristles (thb) on prolaterodistal part; cymbium not fused with bulb, with scattered setae; bulb pear shaped, basal-ventral area bulged, about twice as long as cymbium, stout, tapering apically; embolus system (Fig. 2E–G) complicated, with a wide, ear-shaped protrusion (esp) prolaterally, surface of the protrusion bearing numerous spinules, with a filiform, long and mesially curved projection (ffp) and a narrow branch (nb) retrolaterally.

Female: as in male except as noted. Habitus as in Fig. 3A, B. Slightly larger than male. Body length 1.78; carapace 0.73 long, 0.62 wide; abdomen 1.07 long, 0.69 wide. Abdomen: dorsal scutum covering about 3/4 of abdomen, about 2/3 of abdomen width (Fig. 3A). Sternum without characteristic setae. Postepigastric scutum short, boat-shaped, posterior margin smoothly curved, not fused to epigastric scutum (Fig. 3G).

Female genitalia: ventral view (Fig. 3H, I): posterior margin of epigastric scutum with two dark brown knobs (dkn); surface without external features. Dorsal view (Fig. 3J): with a elliptical posterior receptacle (pr), extending anterior by a narrow, stick-like sclerite (sls); with very short apodemes (apo).

##### Distribution.

Known only from the type locality.

#### Bannana parvula sp. n.

Taxon classificationAnimaliaAraneaeOonopidae

http://zoobank.org/29B780CE-957D-4DD2-ADDC-6083FB3AAFD0

##### Type material.

**Holotype:** male (IZCAS Ar-25067), China: Yunnan Province, Mengla County, Menglun Nature Reserve, Secondary tropical seasonal moist forest (21°54.607'N, 101°17.005'E, Alt: 633 m), pitfall traps, 16–31 March 2007, G. Zheng and Z. Chen leg. **Paratypes:** 1 female (IZCAS Ar-25071), searching, same data as holotype; 1 male (IZCAS Ar-25068), Primary tropical seasonal rainforest (21°57.445'N, 101°12.997'E, Alt: 744 m), searching, 19–25 December 2006; 1 female (IZCAS Ar-25066), Secondary tropical seasonal moist forest (21°54.718'N, 101°16.940'E, Alt: 645 m), pitfall traps, 16–31 March 2007; 1 female (IZCAS Ar-25070), Rubber-tea plantation (21°55.551'N, 101°16.923'E, Alt: 561 m), searching, 19–26 May 2007; 1 female (IZCAS Ar-25069), Rubber plantation (21°54.684'N, 101°16.319'E, Alt: 585 m), searching, 5–12 January 2007.

##### Etymology.

The specific name is Latin, “parv-” = small, referring to the very small body size of this species.

##### Diagnosis.

Males of the new species are similar to those of *Dysderoides kanoi* Grismado & Deeleman, 2014, but can be distinguished by the small size and the ear-shaped protrusion on distal part of bulb (compare Fig. 6A–C and Grismado et al. 2014: fig. 10G–I); females can be distinguished from *Bannana crassispina* sp. n. by the large dorsal abdominal scutum and the rectangular postepigastric scutum (Fig. 5E, I).

##### Description.

Male. Body yellow, legs lighter. Habitus as in Fig. 4A. Body length 1.07; carapace 0.51 long, 0.38 wide; abdomen 0.62 long, 0.34 wide. Carapace oval, *pars cephalica* almost flat in lateral view, dorsal surface smooth; sides reticulated; lateral margin rebordered; no eye remnants visible (Fig. 4B, C). Mouthparts: chelicerae straight, paturon inner margin unmodified; labium anterior margin deeply incised (ldi) (Fig. 4E); endites slender, distally only slightly branched. Sternum: setae sparse, light, needle-like, evenly scattered. Abdomen: dorsal scutum covering full length of abdomen, no soft tissue visible from above, not fused to epigastric scutum. Pedicel tube short, unmodified. Book lung covers round, surface smooth. Postepigastric and epigastric scutum fused, apodemes absent, posterior spiracles connected by groove (Fig. 4F). Leg spines: tibiae I, II with 3 pairs of ventral spines each; metatarsi I, II with 2 pairs of ventral spines each, legs III and IV without spines.

Male genitalia: epigastric region (Fig. 4F) with sperm pore small, oval, rebordered, situated between anterior and posterior spiracles. Palp (Fig. 4H–J): pale-orange; femur slightly enlarged, attached to patella basally; cymbium not fused with bulb, with scattered setae; bulb pear shaped, about twice as long as cymbium, stout, tapering apically; embolus system (Fig. 6A–C) complicated, with a narrow, ear-shaped protrusion (esp) prolaterally, surface of the protrusion bearing numerours spinules, with a filiform, long and mesially curved projection (ffp) and a narrow branch (nb) retrolaterally.

Female: as in male except as noted. Habitus as in Fig. 5A, B, E, F. Body length 1.12; carapace 0.50 long, 0.39 wide; abdomen 0.65 long, 0.32 wide. Abdomen: dorsal scutum covering about 5/6 of abdomen, about equal to the abdomen width (Fig. 5E). Postepigastric scutum rectangular, posterior margin nearly straight, not fused to epigastric scutum (Fig. 5G).

Female genitalia: ventral view (Figs 5I, J, 6D): posterior margin of epigastric scutum with two dark brown knobs (dkn); surface without external features. Dorsal view (Figs 5K, 6E): with a nearly round posterior receptacle (pr), extending anterior by a narrow, stick-like sclerite (sls); with short apodemes (apo).

##### Distribution.

Known only from the type locality.

## Supplementary Material

XML Treatment for Bannana

XML Treatment for Bannana crassispina

XML Treatment for Bannana parvula
